# Short term hypothyroidism affects ovarian function in the cycling rat

**DOI:** 10.1186/1477-7827-8-14

**Published:** 2010-02-11

**Authors:** María Belén Hapon, Carlos Gamarra-Luques, Graciela A Jahn

**Affiliations:** 1 Laboratorio de Reproducción y Lactancia, IMBECU-CONICET, Mendoza, Argentina; 2 Instituto de Ciencias Básicas, Universidad Nacional de Cuyo, Mendoza, Argentina; 3 Instituto de Embriología e Histología, IHEM-CONICET, Facultad de Ciencias Médicas, Universidad Nacional de Cuyo, Mendoza, Argentina

## Abstract

**Background:**

Rats made hypothyroid with propilthyouracil start showing abnormal cycling on the second cycle after the start of the treatment, with a high proportion of spontaneous pseudopregnancies and reduced fertility.

**Methods:**

To investigate some of the mechanisms involved in these reproductive abnormalities, hypothyroidism was induced in virgin rats by propilthyouracil (0.1 g/L in the drinking water) and we determined circulating hormones by radioimmunoassay and whole ovary expression of ovarian hormone receptors, growth factors and steroidogenic enzymes using semi-quantitative RT-PCR.

The study was performed on days 6 to 9 of treatment, corresponding to diestrus I (at 20.00-22.00 h), diestrus II (at 20.00-22.00 h), proestrus and estrus (both at 8.00-10.00 h and 20.00-22.00 h) of the second estrous cycle after beginning propilthyouracil treatment. Another group of rats was mated on day 8 and the treatment continued through the entire pregnancy to evaluate reproductive performance.

**Results:**

Hypothyroidism increased circulating prolactin and estradiol on estrus 5 to 7-fold and 1.2 to 1.4-fold respectively. Growth hormone and insulin-like growth factor 1 diminished 60 and 20% respectively on proestrus morning. Hypothyroidism doubled the ovarian mRNA contents of estrogen receptor-beta on proestrus and estrus evenings, cyp19A1 aromatase mRNA on estrus evening and of growth hormone receptor on proestrus evening. Hypothyroidism did not influence ovulation rate or the number of corpora lutea at term, but a diminished number of implantation sites and pups per litter were observed (Hypothyroid: 11.7 +/- 0.8 vs. Control: 13.9 +/- 0.7).

**Conclusions:**

Short term hypothyroidism alters normal hormone profile in the cycling rat increasing the expression of estrogen receptor-beta and cyp19A1 aromatase on estrus, which in turn may stimulate estradiol and prolactin secretion, favouring corpus luteum survival and the subsequent instauration of pseudopregnancy.

## Background

Hypothyroidism has a prevalence of 0.2 and 2% in male and female populations respectively [1], and if left untreated has severe consequences on the metabolism and function of various organs and systems of the organism, among them the reproductive system [2]. It has been associated with menstrual abnormalities, anovulation and hyperprolactinemia [3], which produce ovulation disorders and reduced fertility in women of childbearing age, with increased risk of miscarriage, premature delivery, placental abruption and poor perinatal outcome with low birth weight [4-7]. Similar deleterious effects of hypothyroidism on fertility are also observed in many mammals [8,9]. The female reproductive cycle depends on a complex interaction between the hypothalamus, pituitary and ovary that interact through an endocrine feedback system, regulating hormone secretion and processes such as folliculogenesis, ovulation and conception. Classically, the gonadotrophins, luteinizing hormone (LH) and follicle stimulating hormone (FSH) were considered as the main regulators of follicular proliferation and differentiation. However, more recently a series of substances secreted by the ovary have been found to participate in this regulation. Among them are steroids like 17β-estradiol (E_2_) and progesterone (P_4_), polypeptides such as inhibin, activin and follistatin and growth factors including insulin-like growth factor I (IGF I), epidermal growth factor, antimüllerian hormone, fibroblast growth factor and angiogenic factors [10].

Thyroid hormones (THs) act at all the levels of regulation within the reproductive system. Triiodothyronine (T_3_) and thyroxine (T_4_) are present in follicular fluid [11]. Granulosa cells of antral preovulatory follicles and ovarian stromal cells express thyroid hormone receptors α and β messenger RNA (mRNA) and protein. Thus, it is conceivable that THs play an essential role in ovarian physiology [12].

In adult rats, hypothyroidism apparently does not directly produce sterility but during the first half of gestation it increases embryonic reabsorption, resulting in smaller litters and augmentation of fetal mortality, although it does not seem to affect implantation [13-16]. On the other hand, hypothyroidism reduces fertility, causing estrous cycle irregularities and spontaneous consecutive pseudopregnancies that are considered to be a consequence of the hyper secretion of prolactin (PRL) during proestrus (P) and estrus (E) [17,14].

The abnormal estrous cycles seen in hypothyroid (HypoT) rats have been associated with diminished circulating growth hormone (GH), IGFs and E_2_, and administration of T_4 _to HypoT rats reverts the effect on circulating hormones and normalizes the estrous cycle [18]. However, the administration of GH to thyroidectomized rats did not improve the timing of gestation, the number of reabsorbed fetuses nor the number of pups *per *litter [19]. Thus, the effect of hypothyroidism on female sexual cycles may no be due solely to the alteration of the GH/IGF axis.

Recently we have shown that virgin rats treated with the antithyroid propilthyouracil (PTU) presented irregular cycles, spontaneous pseudopregnancies and altered circulating ovarian hormones and PRL after the third estrous cycle that resulted in mammary development similar to that of midpregnancy. Furthermore, when the rats were mated 8 days after the start of antithyroid treatment they produced smaller litters [14].

To investigate the biochemical mechanisms involved in the reproductive alterations (smaller litters and spontaneous pseudopregnacies) observed in HypoT rats, we studied the early effects of PTU-induced hypothyroidism on the female reproductive axis analyzing its effect on basic reproductive parameters and ovarian function in virgin rats on the second cycle after beginning antithyroid treatment, that is the cycle when the rats were mated on our previous study [14]. We evaluated the early effect of hypothyroidism on the pattern of circulating hormones, the ovarian expression of several hormone receptors, growth factors and steroidogenic enzymes. We also measured the ovulation rate in virgin rats and the number of *corpora lutea*, implantation sites and pups at term to determine if the reduced litter size of HypoT rats is associated with ovulation failure or with postovulatory phenomena.

## Methods

### Animals

Adult female Wistar rats bred in our laboratory, 2-3 months old, weighing 200-230 g at the onset of treatment and with regular 4 day cycles were used. Vaginal smears from all animals were obtained daily to monitor the estrous cycle. The rats were kept in a light (lights on 6.00 - 20.00 h) and temperature (22-24°C) controlled room. Rat chow (Cargill, Cordoba, Argentina) and tap water or PTU solution were available *ad libitum*. Animal maintenance and handling was performed according to the NIH guide for the Care and Use of Laboratory Animals (NIH publication N8 86-23, revised 1985 and 1991) and the UK requirements for ethics of animal experimentation (Animals Scientific Procedures, Act 1986).

### Experimental design

Hypothyroidism was induced by administration of PTU at a concentration of 0.1 g/l in the drinking water. The treatment was started on E day. To determine the pattern of hormonal secretion and the function of the ovary, groups of 8-10 HypoT or 8-10 control rats were killed by decapitation during the second cycle after initiation of the treatment, starting at diestrus (D) I and at times selected so as to cover most of the stages of the estrous cycle, such as the initiation (D I at 20.00-22.00 h) and end of the luteal phase (D II at 20.00-22.00 h), the peak of estrogen surge on P morning (8.00-10.00 h), the preovulatory peak on P evening (20.00-22.00 h) and the postovulatory phase (E morning at 8.00-10.00 h and evening at 20.00-22.00 h). The denominations of the groups correspond to the time which the animals were sacrificed. Trunk blood was collected and serum separated by centrifugation and stored at -20°C until used for subsequent determination of hormonal parameters. The ovaries were rapidly removed, washed in a cold saline solution, snap-frozen in liquid nitrogen and stored at -80°C until they were used for RNA preparation. To determine the ovulation rate, the oviducts were also removed from the rats sacrificed on E morning and the *ampullae *were isolated. The oocytes were harvested by applying gentle pressure to both ends of the *ampulla*, placed on a slide in phosphate buffer saline and counted under a stereomicroscope.

Another group of 8 rats were mated on the second P (8 days) after starting the treatment. The presence of spermatozoa in the vaginal smears the morning after caging with a fertile male in P night was indicative of mating and this day was counted as day 0 of pregnancy. Two or three days before delivery the rats were caged individually. The day and hour of delivery and the number of pups were recorded. Another group of gestating rats were killed by decapitation on day 21 of gestation and the number of implantation sites of pups *in utero *and of *corpora lutea*, in the ovaries, were recorded.

### Hormone determinations

PRL, LH, FSH, GH and thyroid stimulating hormone (TSH) were measured by double antibody radioimmunoassay using materials generously provided by Dr Parlow and the NHPP (National Hormone and Pituitary Program, Harbor-UCLA Medical Center, Torrance, CA, USA) as previously described [14]. For each determination, all the samples were measured by duplicate in the same assay, and the intra-assay coefficient of variance was less than 10%

E_2_, P_4_, T_3_, T_4 _and IGF 1 concentrations in sera were measured in duplicate by radioimmunoassay using commercial kits for total hormones DSL-4800, DSL-3400, DSL-3100, DSL-3200 and DSL 2900 double antibody radioimmunoassay, respectively; all from Diagnostic Systems Laboratories, Webster, TX). Inter- and intra-assay coefficients of variation were less than 10%.

### RNA isolation and semi-quantitative RT-PCR analysis

Total ovarian RNA was prepared using TRIZOL Reagent (Invitrogen Life Technologies), following the manufacturer's instructions for RNA isolation. Ten micrograms of total RNA were reverse transcribed at 37°C using random hexamer primers and Moloney murine leukemia virus retrotranscriptase (Invitrogen/Life Technologies) in a 20 μl reaction mixture. Aliquots of the reverse transcription reaction mix complementary DNA (cDNA) corresponding to different quantities of cDNA for each reaction were amplified with primers specific for the rat and in the conditions described in Table 1. The conditions and quantities of cDNA added were such that the amplification of the products was in the exponential phase and the assay was linear with respect to the amount of input cDNA. The reactions were carried out with the following cyclic parameters for *cyp19A1 *aromatase and IGF receptor, type I (IGFIR): 95°C for 1 min, 56°C 1 min and 72°C 1 min; IGF BP-5: 92°C 1 min., 58°C 1 min. and 72°C 1 min.; S16, long PRL receptor (PRLR_*long*_), GH receptor (GHR), estrogen receptor alpha (ERα), ERβ: 95°C 1 min., 65°C 1 min. and 72°C 1 min.; IGF 1, IGF BP-3: 95°C 1 min., 62°C 1 min. and 72°C 1 min. All the reactions were terminated with a 5 min. extension at 72°C. RNA samples were assayed for DNA contamination by performing the different polymerase chain reactions (PCR) without prior reverse transcription. The PCR products were analyzed on 1.5% agarose gels containing 0.5 mg/ml ethidium bromide and photographed using a Kodak DC 290 Zoom Digital Camera. Band intensities of semi-quantitative reverse transcription PCR (RT-PCR) products were quantified using NIH Image software. Relative levels of mRNA were expressed as the ratio of signal intensity for the target genes relative to that for the housekeeping gene rat ribosomal protein S16 cDNA.

**Table 1:** Primer sequences and reaction conditions used in the PCR amplification of the various cDNAs

Gene	Sense primer (5'-3')	Anti-sense primer (5'-3')	Added cDNA (ng)	No. of cycles	Gene bank accession N°	Product size (pb)	Ref.
IGF I	AAAATCAGCAGTCTTCCAAC	AGATCACAGCTCCGGAAGCA	50	25	[GenBank:X06108]	299	[49]
S16	TCCAAGGGTCCGCTGCAGTC	CGTTCACCTTGATGAGCCCATT	100	22	[GenBank:XM_341815]	100	[50]
IGFBP3	GCCGCGGGCTCTGCGTCAACGC	CTGGGACTCAGCACATTGAGGAAC	200	25	[GenBank:NM_012588]	415	[49]
IGFBP5	TTGCCTCAACGAAAAGAGC	AGAATCCTTTGCGGTCACA	100	25	[GenBank:NM_012817]	377	[49]
GHR	GAGGAGGTGAACACCATCTTGGGC	ACCACCTGCTGGTGTAATGTC	100	25	[GenBank:J04811]	534	[50]
ERα	AATTCTGACAATCGACGCCAG	GTGCTTCAACATTCTCCCTCCTC	200	25	[GenBank:X061098]	344	[50]
ERβ	AAAGCCAAGAGAAACGGTGGGCAT	GCCAATCATGTGCACCAGTTCCT	100	25	[GenBank:U57439]	352	[50]
PRLLong	AAAGTATCTTGTCCAGACTCGCTG	AGCAGTTCTTCAGACTTGCCCTT	100	30	[GenBank:M74152]	279	[50]
IGFIR	TCCACCATAGACTGGTCTCT	ACGAAGCCATCTGAGTCACT	50	30	[GenBank:L29232]	433	[50]
P450arom	TGCACAGGCTCGAGTATTTCC	ATTTCCACAATGGGGCTGTCC	100	30	[GenBank:M33986]	271	[29]

### Statistical analysis

Statistical analysis was performed using two-way analysis of variance followed by the Bonferroni *post hoc *test to compare any two individual means or using Student's t-test when only two groups were compared [20]. When variances were not homogeneous we performed log transformation of the data. Differences between means were considered significant at the P < 0.05 level.

## Results

### Effect of PTU treatment on serum concentrations of thyroid, pituitary and ovarian hormones and IGF 1

**Figure 1:**
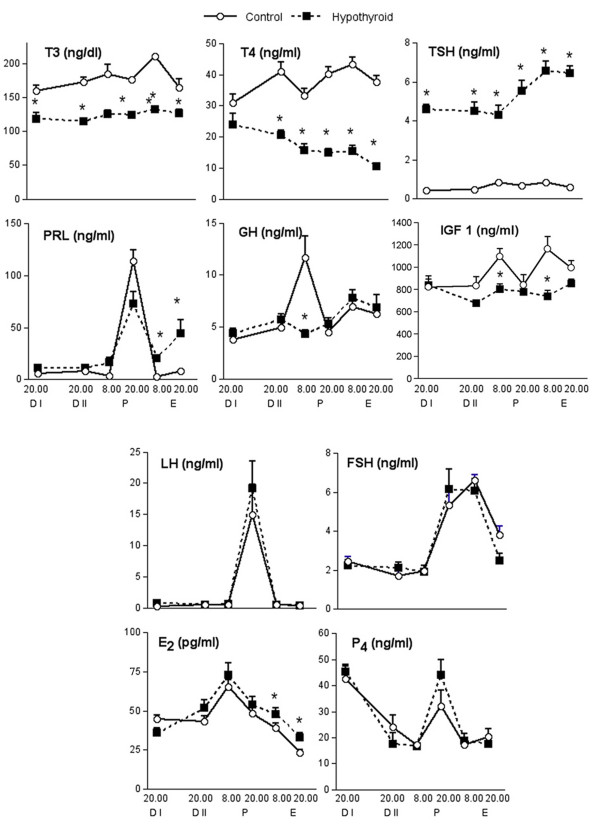
**Circulating hormone concentrations during the estrous cycle in control and HypoT rats during the 2^nd ^estrous cycle after initiation of PTU treatment (0.1 g/l in the drinking water)**. See Materials and Methods section for further details. The results represent the means ± SEM of groups of 6-9 rats. * p < 0.05 compared with respective control group.

PTU treatment was effective in inducing hypothyroidism, as shown by the diminished levels of T_3 _and T_4 _and elevated levels of TSH (Figure 1).

A classical preovulatory pattern of gonadotrophin secretion was observed in both groups and no differences between groups were detected during the estrous cycle in LH or FSH (Figure 1), suggesting that PTU treatment did not affect the pituitary secretion of these hormones. On the other hand, although the preovulatory peak of PRL was normal in the HypoT rats, we observed a significant increase in PRL concentrations during E (Figure 1). Since GH secretion is also influenced by THs, we also measured circulating GH during the estrous cycle. Control rats showed a GH peak on P morning. This peak was not observed in the HypoT rats (Figure 1). Similarly, circulating IGF 1 in control rats peaked on P and E mornings, and hypothyroidism annulled these increases.

To evaluate the effect of hypothyroidism on the ovary we also measured circulating E_2 _and P_4 _during the estrous cycle in the rat. Hypothyroidism did not modify serum P_4 _pattern (Figure 1). As expected both groups showed increased levels during D I (luteal phase) and during the preovulatory surge at P 20.00 h. E_2 _showed a progressive increase from D I until P morning and then diminished after ovulation. In HypoT rats the postovulatory decrease was less prominent, resulting in significantly increased concentrations during E.

### RT-PCR analysis of the relative expression of factors related to the ovarian response to E_2_, PRL and gonadotrophins during the estrous cycle

Since in the HypoT rats PRL and E_2 _levels were increased during E (Figure 1), we evaluated the ovarian response to these hormones determining the expression of PRLR_*long*_, ERα and ERβ during the estrous cycle.

**Figure 2:**
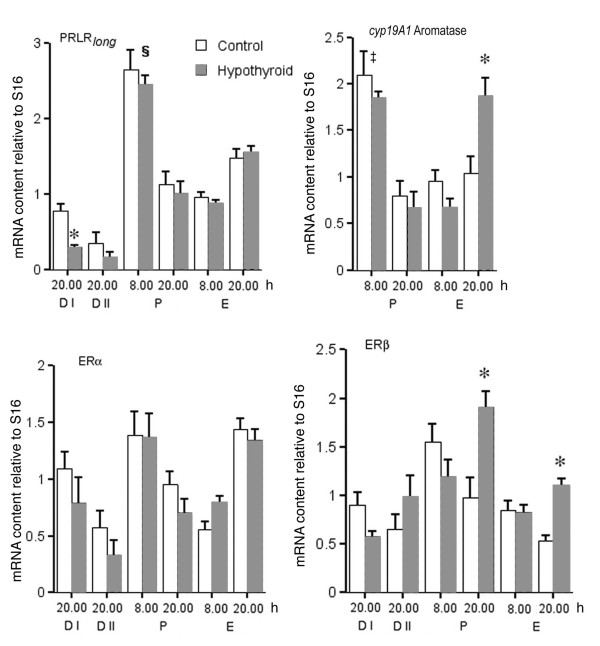
**Ovarian mRNA content relative to S16 (see Materials and Methods section for further details) of PRLR_*long*_, ERα, ERβ and *cyp19A1 *aromatase estimated by semi-quantitative RT-PCR in control and HypoT rats during the 2^nd ^estrous cycle after initiation of PTU treatment**. The results represent the means ± SEM of groups of 6-9 rats. * p < 0.05 compared with respective control group; § p < 0.05 compared with D 1 20.00 h; ‡ p < 0.05 compared with E 8.00 h.

A surge in PRLR_*long *_expression was observed (Figure 2) on P 8.00 h, followed by a second increase on E 20.00 h. The only effect of hypothyroidism was a diminished expression on D I.

Expression of ERα showed a pattern similar to that observed for PRLR_*long*_, with two peaks on P morning and E afternoon and a decrease on E 8.00 h. Hypothyroidism did not modify this pattern significantly (Figure 2).

In control rats, ERβ mRNA content (Figure 2) was elevated on P 8 h, decreased progressively during P afternoon and E and remained low on both D days. In HypoT rats, the highest expression was observed on P 20.00 h, being significantly higher with respect to controls, and a second increase with respect to the controls was observed on E 20.00 h (Figure 2).

In order to determine the ovarian response to the circulating FSH and LH and to explore the cause of the increased circulating E_2 _observed in E (Figure 1), we measured *cyp19A1 *aromatase expression on P and E. In control rats the maximum expression was observed on P 8.00 h, followed by low values on P evening and on E (Figure 2). In HypoT rats the pattern was similar on P and E morning, but on E 20.00 h *cyp19A1 *aromatase expression was significantly higher compared to controls.

### RT-PCR analysis of GH/IGF 1 system relative expression in the ovary during the estrous cycle

**Figure 3:**
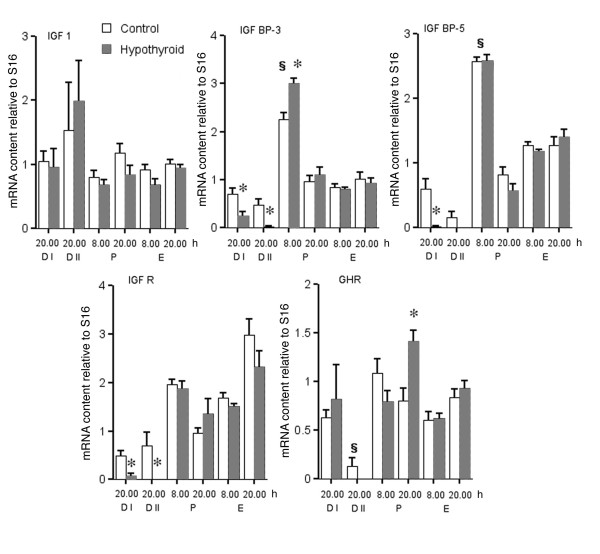
**Ovarian mRNA content relative to S16 of IGF 1, IGF R, IGF BP-3, IGF BP-5 and GHR estimated by semi-quantitative RT-PCR in control and HypoT rats during the 2^nd ^estrous cycle after initiation of PTU treatment**. The results represent the means ± SEM of groups of 6-9 rats.* p < 0.05 compared with respective control group; § p < 0.05 compared with D 1 20.00 h.

Based on the HypoT effects observed on circulating GH, IGF 1 and E_2 _we evaluated the expression, relative to S16, of several components of the GH/IGF axis during the estrous cycle at ovarian level. IGF 1 content did not vary during the estrous cycle and was not affected by hypothyroidism (Figure 3). In contrast, there were significant differences in the expression of IGF binding proteins (IGF BPs). IGF BP-3 content was highest on P 8.00 h (Figure 3), and showed the lowest values on both D days. The same pattern was amplified in the HypoT rats that had significantly higher values than controls on P 8.00 h and significantly lower values on D I and II (Figure 3).

IGF BP-5 showed a pattern similar to that of IGF BP-3, with the highest values on P morning and lowest on D days (Figure 3). The only effect of hypothyroidism on IGF BP-5 content was a diminution on D I (Figure 3).

The expression of IGFIR in control rats (Figure 3) was low on the D days, increased on P 8.00 h, diminished the same day at 20.00 h, and then rose progressively reaching maximum values on E 20.00 h. PTU treated rats had a similar pattern, but showed diminished expression on both D days.

In control rats GHR expression was stable during most of the estrous cycle, except on D II, where there was a marked decrease. HypoT rats had a similar pattern, except for an increase in the expression on P 20.00 h (Figure 3).

### Effect of PTU treatment on the ovulation rate in virgin rats and the number of corpora lutea and implantation sites at the end of gestation

**Figure 4:**
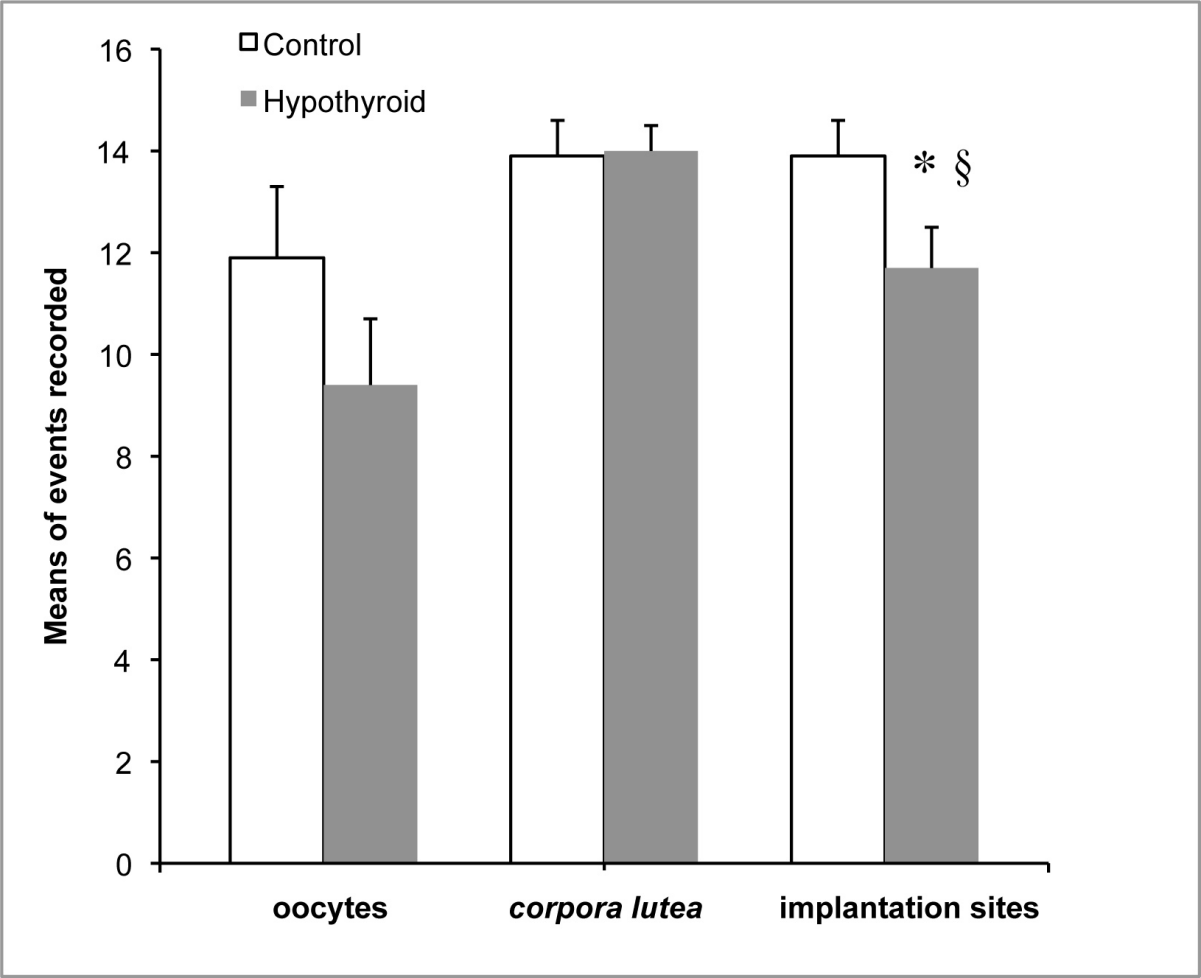
**Number of oocytes, corpora lutea and implantation sites in control and HypoT rats at the end of gestation**. The results represent the means ± SEM of groups of 6-9 rats. * p < 0.05 compared with respective control group; § p < 0.05 compared with corpora lutea counted in the HypoT group.

We have previously shown that HypoT rats have smaller litters [14]. To determine if this is a consequence of an ovulatory deficit, the ovulation rate (measured as number of oocytes *per *rat in the oviduct on E morning) was recorded in control and HypoT rats after 9 days of PTU treatment. HypoT rats had a slightly lower ovulation rate compared to controls, but the difference was not statistically significant (Figure 4).

Next, we evaluated if the diminished number of pups *per *litter was due to a defect in implantation or gestational development, by counting the implantation sites, pups and *corpora lutea *in a group of HypoT and control rats at term. There were no differences in the number of *corpora lutea *but the number of implantation sites and of pups *per *rat was significantly lower in HypoT rats compared with controls (Figure 4). No pup mortality was observed *in utero*, however, the number of implantation sites in HypoT rats was significantly lower than the number of *corpora lutea *(Figure 4).

## Discussion

Hypothyroidism is frequently associated with indirect increases in circulating PRL that in turn increase the risk of anovulation in women. Similarly, in the present work we observed increased circulating PRL on the last day of PTU treatment, corresponding to E, which may be caused by the previously described increase in thyrotropin releasing hormone (TRH) provoked by the HypoT state [21] and to the increased circulating E_2 _observed simultaneously. TRH has a potent stimulating action on pituitary PRL secretion in the presence of elevated levels of E_2 _[22] and E_2 _*per se *is a potent stimulator of the synthesis and release of PRL. In the rat, hyperprolactinemia reduces the pituitary response to gonadotropin releasing hormone (GnRH), hindering preovulatory LH secretion [23] and makes the hypothalamus more susceptible to stimuli capable of inducing pseudopregnancies [24].

The effects of hypothyroidism on the gonadotrophic axis are contradictory, some authors have found that PTU treated rats had diminished concentration of LH on D and P but the preovulatory surge was conserved [25], while others describe a reduced proportion of ovulating rats, but increased preovulatory levels of LH in those rats that ovulated after radiochemical thyroidectomy with ^131^I [17]. We found no significant variations on preovulatory LH levels on P, the eighth day after initiation of PTU treatment. Most probably, the discrepancies between these three studies may be a consequence of the different treatment paradigms used to induce hypothyroidism. We did not find, either, any effect on the ovulation rate, indicating that at this early stage of PTU treatment, the gonadotrophic axis does not seem to be affected.

On the other hand, the increased serum PRL observed in E may be responsible for the induction of spontaneous pseudopregnancies that we observed after more prolonged PTU treatments [14]. Other authors also have found that hypothyroidism induces prolonged D accompanied with elevated circulating P_4 _indicative of pseudopregnancy [17,25]. E_2 _regulates luteal function facilitating the uptake of cholesterol for P_4 _synthesis [26]. Thus, the elevated E_2 _and PRL observed in E may stimulate subsequent P_4 _synthesis and secretion in the newly formed *corpora lutea*, which in turn triggers the semi-circadian secretion of PRL necessary to maintain luteal function and the pseudopregnant state [27].

The increased serum concentration of E_2 _observed during E may be the result of a permissive action of PRL that augments the number of luteal LH receptors. This favours gonadotrophic action that in turn promotes the conversion of P_4 _into androstenodione and then to E_2_, a mechanism that has been described in the corpus luteum of the rat [28]. Bao *et al*. [29] suggest that E_2 _stimulates expression of *cyp19A1 *aromatase through binding to ERβ in granulosa and luteal cells. Thus, we may infer that the increased expression of *cyp19A1 *aromatase on E is consequence of the elevated circulating E_2 _acting on elevated ERβ that in turn stimulates E_2 _synthesis and PRL release. In this sense, a positive feedback loop may be established leading to a sustained increase in circulating PRL and E_2 _during E.

Rat ovaries express both short and long isoforms of the PRLR, although the long one, PRLR_*long*_, is the most abundant and in agreement with our result, its expression surges on P [30]. Both isoforms are expressed in the *corpora lutea*, granulosa and the interstitial cells adjacent to the Graafian follicles, although in E and D I the long isoform is majoritarily expressed on luteal tissue [30]. ERs also are expressed in rat ovaries. ERα is expressed in low levels in different types of ovarian cells and in the oocyte, but it is not expressed in mature follicles nor in the *corpora lutea *[31,32]. ERβ is more highly expressed in ovarian tissues, mostly in granulosa cells of the developing follicles and to a lesser extent in the newly formed *corpora lutea *[31,32]. It has also been shown that PRL, acting through PRLR_*long *_stimulates ERα and ERβ mRNA expression in the *corpora lutea *and in primary culture of rat granulosa cells [33]. Although PRLR_*long *_mRNA content was not modified by hypothyroidism, the elevated circulating PRL concentrations may be responsible for the high ERβ mRNA content found on E.

On the other hand, since the increase in ERβ mRNA was observed on P, before the increase in PRL and E_2_, it may be a direct effect of hypothyroidism and have a role in the subsequent increase in circulating E_2 _and PRL. Thus, the increase in ERβ may have increased the ovarian response to circulating E_2_, which in turn stimulates *cyp19A1 *aromatase expression and E_2 _synthesis. These data suggest that the negative feedback exerted by E_2 _may be partially blocked in HypoT rats, sensitizing the ovary to E_2 _actions during P and E. Since it has been largely demonstrated that, in the rat, E_2 _stimulates steroidogenesis and luteal hypertrophy and synergizes with the effect of PRL in the ovary, these factors may promote *corpus luteum *survival favouring the establishment of pseudopregnancy [26,34-37].

Since during E, *cyp19A1 *aromatase is almost exclusively expressed in the *corpus luteum *[38], the increased expression observed in the HypoT rats may be responsible for the increased levels of circulating E_2 _observed at the same time.

Hypothyroidism also diminishes synthesis and secretion of GH in the rat [39]. In particular, the stimulation of GH release produced by E_2_depends on the participation of THs [40]. The GH surge observed on P morning in the control rats may be due to the elevated circulating E_2_, and its absence in the HypoT rats may be directly linked to the low levels of THs, that may have offset the stimulatory action of E_2_. In addition, the low GH levels on P may play a part in the decreased circulating IGF 1 levels on P morning, since GH is the main stimulus for hepatic IGF 1 production, and the liver is the main source of this hormone [41]. The decreased levels of IGF 1 on E morning, in contrast, may be a direct effect of hypothyroidism.

Numerous investigations have demonstrated actions of GH on steroidogenesis, gametogenesis and gonadal differentiation. Many of these effects are mediated by local actions of IGF 1. It has been postulated that this growth factor has an essential participation in follicular development [42] and ovarian steroidogenesis [10]. GH exerts most of its effects in the ovary through binding to its specific receptor. For example, mice lacking GHR present a diminished ovulation rate, caused by the failure of the ovarian response to gonadotrophins [43]. GH promotes FSH-induced LH receptor (LHR) synthesis, and the administration of IGF 1 does not improve the ovulation rate, suggesting a direct participation of GH in the ovulation process. IGF 1 promotes FSH receptor (FSHR) expression, and in turn FSH augments both IGFIR and FSHR. Besides, this growth factor exerts its action through its own receptor, present in granulosa, thecal and luteal cells. IGFIR expression is positively regulated by FSH, LH and GH [10] and it is expressed in the oocyte, mediating its maturation by IGF 1 [44]. Thus, the increase in GHR on P in the HypoT rats may have compensated for the absent GH surge, allowing follicle maturation to culminate in ovulation.

Bachelot *et al*. [43] reported that IGF 1 produced locally in the ovary is independent of GH. Although our present and previous results [14] showed that hypothyroidism reduces serum levels of this growth factor (mainly of hepatic origin) in virgin rats with prolonged PTU treatment, we did not observe differences between control and HypoT rats in ovarian IGF 1 mRNA, nor between the days of the cycle. It is possible that IGF 1 regulation by THs is tissue specific, but we cannot dismiss the possibility that this lack of effect is due to the short length of the PTU treatment.

IGF BP-3 sequestrates circulating IGF 1, modulating its action. IGF BP-3 is expressed preferentially in ovarian theca and interstitial cells and it may have an antigonadotrophic effect through sequestering of IGF 1. Our results suggest an altered gonadotrophic response by the diminished availability of IGF 1 on P, while on D I and D II, in contrast, the lower IGF BP-3 concentrations may allow IGF 1 to be more available. Fraser *et al*. [45] demonstrated that IGF BP-3 expression in *corpus luteum *is more intense during luteolysis. Thus, the diminished expression observed during D may promote *corpus luteum *survival, favouring the establishment of pseudopregnancy.

IGF BP-5 is the most abundant IGF 1 binding protein in the ovary, mainly expressed in the granulosa cells of atretic follicles, interstitial cells, *corpus luteum *and also in the superficial epithelium [46,47]. Our results show that IGF BP-5 expression was not affected on P, and since in D IGF BP-5 is selectively expressed in superficial epithelium [46], we may assume that its activity is not physiologically relevant at this moment.

Lastly, ovulation rate and the number of *corpora lutea *were not affected significantly by the short term treatment with PTU, suggesting that ovarian failure is not responsible for the diminished number of pups *per *litter previously observed [14]. However, the number of implantation sites and of pups at delivery was smaller than the number of *corpora lutea *in HypoT rats, suggesting that the deleterious effects of hypothyroidism are exerted on events that occur after ovulation. All these data point to impairments in fertilization or diminished capacity of the embryos to implant as a probable cause of the reduced litters born to HypoT mothers.

The factors affecting intrauterine fetal survival and/or implantation that may be responsible for the diminished number of pups *per *litter remain unclear and need further investigation.

Hypothyroid women have increased rates of ovarian failure and ovulatory dysfunctions [4,48]. The data presented in this investigation may contribute to the understanding of the influence of thyroid hormone disorders on infertility and support the checking of thyroid status in patients with reproductive alterations.

## Conclusions

The present results show that during the second estrous cycle after the start of PTU treatment the rats are frankly HypoT and show some alterations in hormone secretion, and in the expression of ovarian receptors, members of the GH/IGF family and *cyp19A1 *aromatase that may stimulate luteal function and survival and thus account for the appearance of spontaneous pseudopregnancies previously observed [14,17,25]. On the other hand, the observed effects of short-term hypothyroidism do not seem to affect significantly the ovulation rate, indicating that the smaller litters born to HypoT rats seem to be caused by postovulatory events not linked to ovulation rate or *corpus luteum *formation.

## List of abbreviations

cDNA: DNA copy; E: estrus; E_2_: estradiol; ER: estrogen receptor; D: diestrus; FSH: follicle stimulating hormone; FSHR: FSH receptor; GH: growth hormone; GHR: GH receptor; GnRH: gonadotropin release hormone; HypoT: hypothyroid; IGFIR: IGF receptor, type I; IGFBP: IGF binding protein; IGF: insulin-like growth factor; LH: luteinizing hormone; LHR: LH receptor; mRNA: messenger RNA; P: proestrus; P_4_: progesterone; PCR: polymerase chain reaction; PRL: prolactin; PRLR: PRL receptor; PRLR_*long*_: long form of the PRLR; PTU: propylthyouracil; RT-PCR: reverse transcription PCR; T_3_: triiodothyronine; T_4_: thyroxine; THs: thyroid hormones; TRH: thyrotropin releasing hormone; TSH: thyroid stimulating hormone

## Competing interests

The authors declare that they have no competing interests.

## Authors' contributions

MBH designed and conceived the study, carried out the experimental animal model, immunoassays, RT-PCR, performed the data analysis and drafted the manuscript. CGL collaborated in processing the samples, RT-PCR and helped draft the manuscript. GAJ participated in the study design and coordination, data analysis and helped draft the manuscript. All authors read and approved the final manuscript.
